# Continuous Beam Steering Through Broadside Using Asymmetrically Modulated Goubau Line Leaky-Wave Antennas

**DOI:** 10.1038/s41598-017-12118-8

**Published:** 2017-09-15

**Authors:** Xiao-Lan Tang, Qingfeng Zhang, Sanming Hu, Yaqiang Zhuang, Abhishek Kandwal, Ge Zhang, Yifan Chen

**Affiliations:** 1 The Department of Electrical and Electronic Engineering, Southern University of Science and Technology, Shenzhen, 518055 China; 2 State Key Laboratory of Millimeter Waves, Southeast University, Nanjing, 210096 China; 3 Air and Missile Defense College, Air Force Engineering University, Xi’an, 710051 China; 4 The Department of Computer Science, University of Waikato, Hamilton, 3240 New Zealand

## Abstract

Goubau line is a single-conductor transmission line, featuring easy integration and low-loss transmission properties. Here, we propose a periodic leaky-wave antenna (LWA) based on planar Goubau transmission line on a thin dielectric substrate. The leaky-wave radiations are generated by introducing periodic modulations along the Goubau line. In this way, the surface wave, which is slow-wave mode supported by the Goubau line, achieves an additional momentum and hence enters the fast-wave region for radiations. By employing the periodic modulations, the proposed Goubau line LWAs are able to continuously steer the main beam from backward to forward within the operational frequency range. However, the LWAs usually suffer from a low radiation efficiency at the broadside direction. To overcome this drawback, we explore both transversally and longitudinally asymmetrical modulations to the Goubau line. Theoretical analysis, numerical simulations and experimental results are given in comparison with the symmetrical LWAs. It is demonstrated that the asymmetrical modulations significantly improve the radiation efficiency of LWAs at the broadside. Furthermore, the measurement results agree well with the numerical ones, which experimentally validates the proposed LWA structures. These novel Goubau line LWAs, experimentally demonstrated and validated at microwave frequencies, show also great potential for millimeter-wave and terahertz systems.

## Introduction

Conventional two-wire transmission lines (TLs), such as coplanar waveguide (CPW) and microstrip TL, are most commonly used as wave guiding structures in microwave and millimeter-wave antennas. However, these two-wire TL antennas suffer from a low radiation efficiency due to the high losses in the dielectric and ground planes^1,2^. This is mainly attributed to the high concentration of electrical field in the lossy dielectric substrate, which leads to an increase of both dielectric and ohmic losses. The problem becomes worse when the frequency increases to the submillimeter-wave or terahertz (THz) bands. One possible solution is to remove the ground planes of such TLs, forming therefore a groundless or single-wire TL, allowing a significant reduction of ohmic and dielectric losses. The single-wire transmission line (TL), firstly introduced by Sommerfeld^3^, supports a non-radiating wave propagating along an infinitely long cylindrical metal wire with finite conductivity. Based on this principle, Goubau proposed a single-wire transmission line with a coated dielectric, which is known as Goubau line^4,5^. The electrical fields are closely confined along the metallic surface, giving rise to a surface wave mode propagation. More research works based on Goubau line have been carried out in 1960s at much higher frequencies^6^. Very recently, researchers have highlighted the propagation of terahertz waves on a single bare metal wire^7^. Thereafter, a planar implementation of the Goubau line on a thin dielectric substrate was widely explored and applied to low-loss transmission lines^8,9^, leaky-wave antennas^10,11^ and so on. Low-loss transmission property of Goubau line has been demonstrated and validated in submillimeter wave frequency band and even in the terahertz frequency band^12,13^. It should be mentioned that corrugating the surface of the metallic strip with periodic slots has a similar effect^14^. Nevertheless, this new TL, called spoof surface plasmon (SSP) TL^15–18^, usually exhibits higher losses than Goubau lines.

The fundamental mode of the aforementioned single-wire TLs, i.e. planar Goubau line and SSP TL, is a slow-wave bounded mode that does not radiate due to the phase mismatch with the wave in the air. In order to make this kind of single-wire structure efficiently radiate power as an antenna, there are two feasible approaches, i.e., exciting the higher fast-wave modes of TLs or introducing deliberately periodic discontinuities/modulations in the guiding structure, which correspond to uniform leaky-wave antennas (LWAs) and periodic LWAs, respectively. Compared with the uniform LWAs, an important advantage of periodic LWAs is the possibility of continuous frequency scanning from backward to forward across the broadside direction^19^, depending on the phase constant used in the excitation. Goubau-line-based^10^ and SSP-TL-based periodic LWAs^20^ have been investigated respectively for beam scanning ability. In the work^10^, a linear dipole array was loaded along the Goubau line as a periodic perturbation to the original mode so that it can radiate into the air. The planar dipole sources were later replaced by the crossed dipoles for exciting circularly polarized leaky waves^11^. Nevertheless, it should be noticed that the gain and radiation efficiency degradation at broadside is a major issue in all periodic LWAs, including Goubau line LWAs in refs^10,11^. One possible explanation of this degradation is that an open stopband phenomenon^1^ occurs at the broadside frequency, which results in a dramatic drop of gain and radiation efficiency. Many efforts have been made to resolve this issue^21–27^. It has been demonstrated that the asymmetry in the LWA structures, either transversal^24,25^ or longitudinal^26,27^, is useful to avoid the performance degradation at the broadside. Nevertheless, there is no related research work published on Goubau-line-based periodic LWAs.

In this paper, we propose the design of periodic Goubau line LWAs by continuously modulating the Goubau line profile, which exhibits more compactness than the dipole loaded structures^10,11^. The energy leakage is produced by adding the periodic modulations of sine-wave profile all through the Goubau line. The radiation pattern of the antenna is omnidirectional because the structure radiates in two symmetric directions due to the absence of the ground plane of the Goubau line. The main beam of the proposed LWAs steer continuously from backward direction to forward direction. In order to improve the radiation efficiency at the broadside, both transversally and longitudinally asymmetrical modulations are explored. The experimental results reveal that the broadside radiation performance, in terms of the efficiencies and the realized gains, of both asymmetrical Goubau line LWAs are significantly improved in comparison with their symmetrical counterparts. These new Goubau line LWAs by periodic modulations are experimentally demonstrated at microwave frequencies which have great potential applications in integrated Goubau line circuits.

## Results

### Goubau line LWAs using periodic modulations

**Figure 1:**
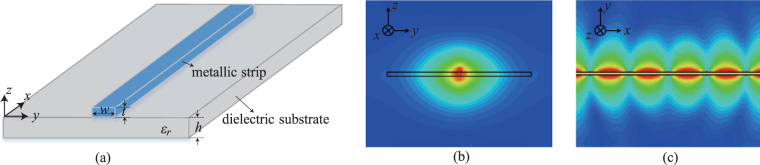
Planar Goubau line on a thin dielectric substrate. (**a**) 3D schematic view; (**b**) The electrical field distribution on the cross-section (*y*–*z* plane). (**c**) The electrical field distribution on the surface of Goubau line (*x*–*y* plane).

The original Goubau line is composed of a cylindrical conductor with a coated dielectric layer^4,5^. The planar implementation of this Goubau line is formed by a thin metallic strip on a dielectric substrate as shown in Fig. 1(a), where the parameters *w*, *t* and *h* denote the line width, the strip thickness and the substrate thickness, respectively. It should be noticed that this configuration is quite similar to the conventional microstrip line except that there is no ground plane at the bottom of the substrate, leading to much lower transmission attenuation. Figure 1(b) and (c) plot the electric field distributions on the cross section (*y*–*z* plane) and on the surface of the line (*x*–*y* plane), respectively. Note that, the electromagnetic fields are highly confined to the metallic strip, which validates that a surface wave is excited for propagation along the Goubau line.

**Figure 2:**
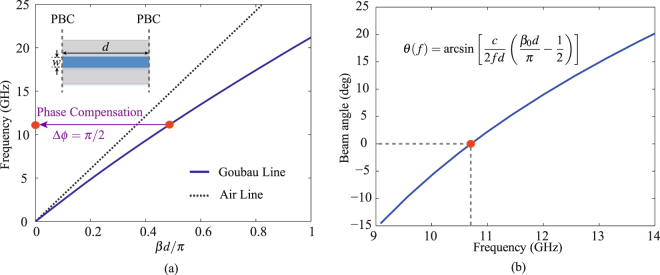
(**a**) Dispersion curve of the planar Goubau line (*w* = 1 mm, *d* = 5.4 mm, *h* = 1.52 mm and *t* = 18 um) on a Rogers 4003 C substrate (*ε*_*r*_ = 3.38, tan*δ* = 0.0027), where *β* is the propagation constant of the Goubau line. (**b**) The predicted beam scanning property of the periodically modulated Goubau line leaky-wave antennas.

The dispersion curve of the planar Goubau line (*w* = 1 mm, *d* = 5.4 mm, *h* = 1.52 mm and *t* = 18 um) on a Rogers 4003 C substrate (*ε*_*r*_ = 3.38, tan*δ* = 0.0027) is plotted in Fig. 2(a). It is calculated by enforcing periodic boundary conditions (PBC) on both sides of the Goubau line using the commercial software CST Microwave Studio, as illustrated by the inset figure of Fig. 2(a). Note that, the dispersion curve of the fundamental mode is always below that of the air. Therefore, it is a slow-wave mode that does not radiate due to the phase mismatch with the wave in the air. For instance, considering a mismatched phase with the broadside radiation Δ*ϕ* of *π*/2 (i.e.*βd* = *π*/2), we deduce that the corresponding frequency related to the broadside direction is 10.7 GHz. To compensate the mismatched phase and hence to excite the radiation, an efficient approach is to periodically modulate the Goubau line. The periodic perturbation of the transmission line leads to an infinite number of space harmonics, whose propagation constants are characterized by

1 $${\beta }_{n}={\beta }_{0}+2n\pi /p,$$

where *p* is the modulation period, *β*_0_ denotes the propagation constant of the unmodulated Goubau line, *β*_*n*_ indicates the propagation constant of the *n* ^th^ space harmonic (*n* = 0, ±1, ±2, …). One may consider the space harmonic *n* = −1 whose propagation constant is *β*_−1_ = *β*_0_ − 2*π*/*p*. To compensate the mismatched phase, Δ*ϕ* = *π*/2 in Fig. 2(a), one has *β*_−1_ *d* = *β*_0_ *d* − 2*dπ*/*p* = 0, which, upon substitution of *β*_0_ *d* = *π*/2, finally leads to *p* = 4*d*. Therefore, if the modulation period is *p* = 4*d*, the modulated Goubau line radiates at the broadside direction at 10.7 GHz.

Let us consider the frequency scanning property of the radiated beam by the periodically modulated Goubau line. For a leaky-wave antenna, the beam direction, *θ*, is computed by *θ* = arcsin(*β*_−1_/*k*_0_) where *k*_0_ is the propagation constant in the air. After applying *β*_−1_ = *β*_0_ − 2*π*/*p* and *p* = 4*d*, one obtains

2 $$\theta =\arcsin (\frac{{\beta }_{0}}{{k}_{0}}-\frac{\pi }{2{k}_{0}d})\mathrm{,\ }$$

which, upon substitution of *k*_0_ = 2*πf*/*c* where *c* is the light speed in the air and *f* is the operational frequency, is further reformulated as

3 $$\theta (f)=\arcsin [\frac{c}{2fd}(\frac{{\beta }_{0}d}{\pi }-\frac{1}{2})]\mathrm{.\ }$$

It is easy to verify by Eq. (3) that the beam angle is zero (corresponding to broadside radiation) at 10.7 GHz, since one has *β*_0_ *d*/*π* = 1/2 at this frequency according to Fig. 2(a). Once the modulation period is determined, further increasing the frequency results in a beam scanning from backward direction to forward direction passing through the broadside direction, leading therefore to a frequency-steerable radiation pattern. Figure 2(b) plots the frequency scanning property of the beam calculated using Eq. (3). The beam angle scans from −14.5° to + 15.1° within the frequency range 9–13 GHz, across the broadside point (*θ* = 0°) at 10.7 GHz. Usually, the leaky-wave antenna suffer from so-called open stopband at the broadside, leading to a radiation efficiency degradation. To overcome this problem, we propose two asymmetrical modulation approaches, which will be introduced in the forthcoming section.

#### Symmetrical & Asymmetrical LWAs: Analysis, Design, simulations and experimental results

**Figure 3:**
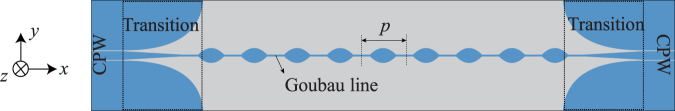
The configuration of a sinusoidally-modulated periodic leaky-wave antenna based on Goubau line.

Figure 3 shows the configuration of the symmetrically modulated Goubau line LWA, where the line profile is modulated by a sinusoidal function. This configuration is very similar to the series-fed patch antenna where the wide lines serve as radiating patches. On both sides of the antenna, tapered ground planes and lines are employed for a smooth transition and mode conversion between 50-Ω coplanar waveguide (CPW) line (width = 4.5 mm, gap = 0.3 mm) and Goubau line. This transition structure efficiently converts the transversal electromagnetic (TEM) mode in CPW to a transversal magnetic (TM) surface-wave mode in Goubau line.

**Figure 4:**
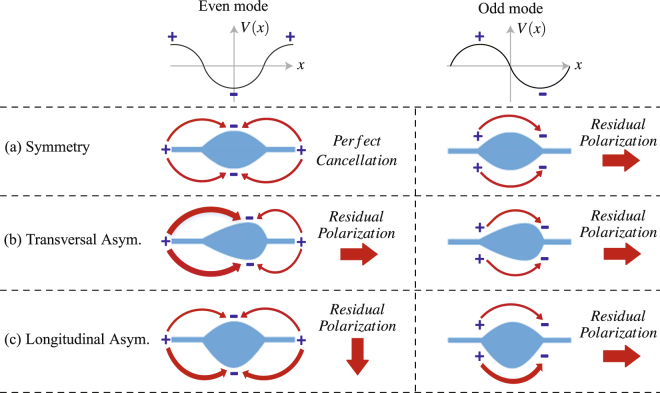
Electric field distributions of (**a**) symmetrical, (**b**) transversal asymmetrical and (**c**) longitudinal asymmetrical configurations under even-mode and odd-mode excitations.

**Figure 5:**
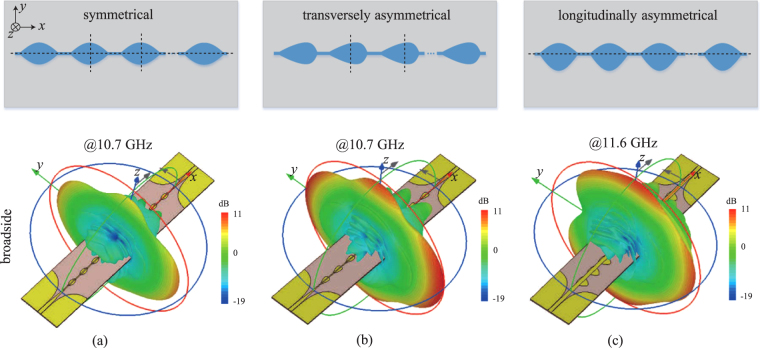
The configurations of symmetrical and asymmetrical Goubau line LWAs and the corresponding 3D radiation pattern at the broadside. (**a**) The symmetrical configuration. (**b**) The transversely asymmetrical configuration. (**c**) The longitudinally asymmetrical configuration.

The sinusoidally-modulated periodic leaky-wave antenna in Fig. 3 exhibits a symmetrical profile both in transversal and longitudinal plane, which usually suffers from a low radiation efficiency at the broadside. Many theoretical works^24–27^ have been reported to analyze this problem. Here, we explain it using even-mode and odd-mode analysis, as shown in Fig. 4. Let us consider the broadside frequency at which the unit cell is one wavelength and the electric voltage distribution *V*(*x*) spans one period across the unit cell. There are two types of *V*(*x*), namely even mode and odd mode, exhibiting cosine and sine profiles, respectively. Assuming the leaky-wave antenna works in a traveling-wave regime and hence exhibits a continuous moving electric voltage profile, the even and odd modes will alternate at different instants. Therefore, for a traveling-wave antenna, both modes should efficiently radiate in order to achieve a high radiation efficiency at the broadside. Figure 4(a) displays the electric fields of the symmetric configuration under both even-mode and odd-mode excitations. Note that, the electric field of the even mode has a perfect cancellation at the broadside direction, and the electric field of the odd mode has a residual radiation polarized in the longitudinal direction. Therefore, the critical reason of the low radiation efficiency at the broadside is due to the even mode exhibiting a field symmetry at both transversal and longitudinal planes. Breaking either of the two symmetries naturally increases the radiation efficiency at the broadside. Here, we propose two configurations in Fig. 4(b) and (c), corresponding to the symmetry breaking in transversal and longitudinal planes, respectively. In the case of transversal asymmetrical configuration, we drag the patch along the longitudinal direction, leading to a residual radiation of the even mode polarized in the longitudinal direction. In the case of longitudinal asymmetrical configuration, we drag the patch along the transversal direction, leading to a residual radiation of the even mode polarized in the transversal direction. Note that, both even and odd modes of the two asymmetrical configurations radiate at the broadside, and hence lead to an improved radiation efficiency. This explains how the symmetry breaking helps to improve the radiation efficiency at the broadside. The determination of the exact configuration requires the full-wave simulation and optimization. To better compare the three Goubau line LWAs, we plot their 3D radiation patterns at the broadside direction in the same scale, as shown in Fig. 5. We can clearly observe that the two asymmetrically modulated LWAs radiate much more energy at the broadside than the symmetrically modulated LWA.

**Figure 6:**
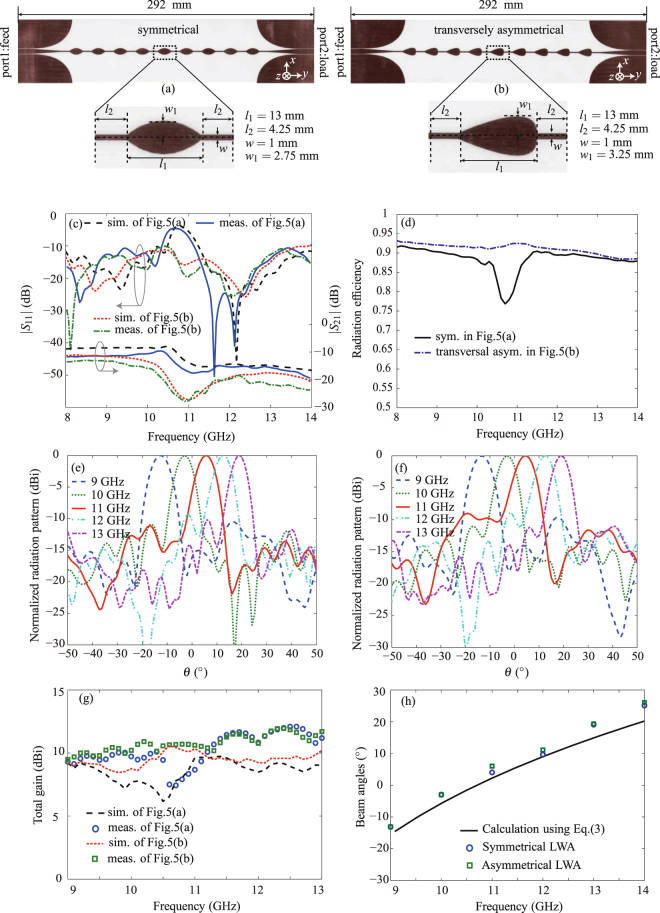
Performance comparison of the transversely symmetrical and asymmetrical LWAs. (**a**) The fabricated prototype of the transversely symmetrical LWA. (**b**) The fabricated prototype of the transversely asymmetrical LWA. (**c**) The simulated and measured scattering parameters of both LWAs. (**d**) The radiation efficiencies of both LWAs. (**e**) The normalized frequency-dependent E-plane radiation patterns of the LWA in Fig. 6(a). (**f**) The normalized frequency-dependent E-plane radiation patterns of the LWA in Fig. 6(b). (**g**) The measured and simulated total gains of both LWAs. (**h**) The calculated and measured radiation beam angels of both LWAs.

**Figure 7:**
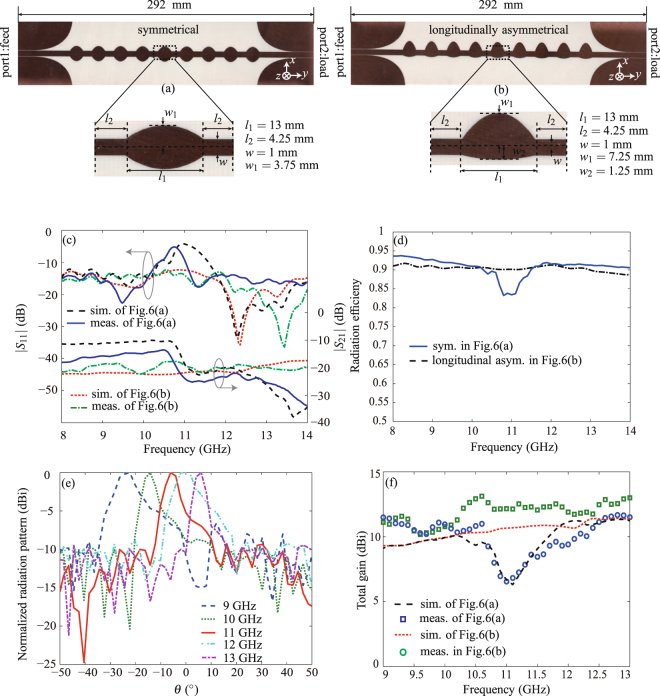
Performance comparison of the longitudinally symmetrical and asymmetrical LWAs. (**a**) The fabricated prototype of the longitudinally symmetrical LWA. (**b**) The fabricated prototype of the longitudinally asymmetrical LWA. (**c**) The simulated and measured scattering parameters of both LWAs. (**d**) The radiation efficiencies of both LWAs. (**e**) The normalized frequency-dependent E-plane radiation patterns of the LWA in Fig. 7(b). (**f**) The measured and simulated gains of both LWAs.

To experimentally validate the proposed periodically-modulated LWAs based on the Goubau line, we design and fabricate both symmetrically and asymmetrically modulated LWAs, as shown in Figs 6(a) and (b), and 7(a) and (b), respectively. All the prototypes are implemented on 1.52-mm-thick Rogers 4003 C substrates (with permittivity 3.38 ± 0.05 and loss tangent 0.0027). The modulation periods are 21.6 mm and the total lengths are all 7*λ*_0_ at 10 GHz.The transversally and longitudinally asymmetrical LWAs are compared with the symmetrical LWA in Figs 6 and 7, respectively. One should note that, the longitudinally symmetrical/asymmetrical LWAs [in Fig. 7(a) and (b)] employ wider Goubau lines than transversally symmetrical/asymmetrical LWAs [in Fig. 6(a) and (b)] for better impedance matching and reflection suppression. Since Goubau line with a different width exhibits a different dispersion curve and hence a different wavelength, the broadside frequency of the longitudinally asymmetrical LWA is different from that of the transversally asymmetrical LWA. This explains why we plot the 3D radiation patterns at 10.7 GHz in Fig. 5(b) but at 11.6 GHz in Fig. 5(c).

Figure 6(c) shows the measured and simulated scattering parameters for both transversally symmetrical and asymmetrical LWAs. The measured responses agree well with the simulated results, despite of a slight frequency shift possibly due to the tolerance in dielectric permittivity (3.38 ± 0.05). The measured *S*_21_ responses for both structures are below −10 dB within 8–14 GHz, which indicates that both LWAs are long enough to radiate out most of the input power. Note that, the measured *S*_11_ of the symmetrically modulated LWA in Fig. 6(a) is lower than −10 dB within 8–14 GHz except a small frequency range around 10.7 GHz, corresponding to the broadside radiation as shown in Fig. 5(a). This open stopband leads to a performance drop in both radiation efficiency (from 92% to 77%) and gain (from 11.0 dBi to 7.2 dBi), as shown in Fig. 6(d) and (g), respectively. In contrast, the transversally asymmetrical LWA in Fig. 6(a) completely suppresses the open stop band, achieving a stable reflection below −10 dB and flat radiation efficiency and total gain within the whole frequency band 8–14 GHz, as shown in Fig. 6(c), (d) and (g). Therefore, the transversally asymmetrical profile helps to improve the radiation efficiency and gain at the broadside. Figure 6(e) and (f) show the measured frequency-dependent E-plane radiation patterns of the symmetrical and asymmetrical LWAs, respectively. Figure 6(h) summarizes their frequency-dependent beam angles in comparison with the theoretical prediction using Eq. (3). Note that, the two beams continuously scan from *θ* = −13.1° to *θ* = 19.1° through the broadside within 9–13 GHz. Also, the two beam angles, closely following the calculated curve, are almost identical within the whole frequency band. Therefore, breaking the transversal symmetry does not changing the beam scanning.

For the comparison of longitudinally symmetrical and asymmetrical LWAs in Fig. 7, a similar phenomena is observed. The symmetrical LWA in Fig. 7(a) exhibits an open stopband with high *S*_11_ around 11.6 GHz [as shown in Fig. 7(c)], and hence a performance drop in both radiation efficiency [as shown in Fig. 7(d)] and total gain [as shown in Fig. 7(f)], whereas the asymmetrical LWA in Fig. 7(b) maintains a low reflection and a flat radiation efficiency and gain within the overall operation frequency band 9–13 GHz. Figure 7(e) also shows the measured E-plane radiation patterns at different frequencies for the asymmetrical LWA, which exhibits a beam shift compared with the radiation pattern of the transversally asymmetrical LWA in Fig. 6(f). This is due to the fact that a wider Goubau line with a longer wavelength is used in longitudinally asymmetrical LWA for better impedance matching.

## Discussion

We designed and compared four periodic Goubau line LWAs using both symmetrical and asymmetrical modulations. These LWAs offer the possibility of continuous beam scanning from the backward direction to the forward direction through the broadside within 9–13 GHz. It has been shown that the symmetrical LWA suffered from the radiation performance drop at the broadside direction, which was improved by both transversally and longitudinally asymmetrical modulations. The measurement results have a good agreement with the simulations, enabling the broadband frequency scanning application in radar and communication systems at microwave frequencies where large phased-array beam scanning is required. Note from Figs 6(e,f) and 7(e) that both symmetrical and asymmetrical Goubau line LWAs have poor side lobe levels at some frequencies due to the uniform unit cells employed in the design. One feasible approach to improve the side lobe levels is using non-uniform unit cells to redistribute the power. Also, with the proper scaling-down of the Goubau line physical dimensions, the antennas working frequency can be extended to submillimeter or even terahertz bands. Benefiting of the low-loss transmission property of the Goubau line as the frequency increases, high-performance THz LWAs can probably obtained. In addition, associated with the development of advanced silicon technologies (e.g. CMOS technologies), more applications such as THz medical imaging systems with the integrated THz LWAs may be investigated.

## Methods

The electromagnetic simulations of the LWAs are performed by using the commercial software CST Microwave Studio. To analyze the dispersion property of the Goubau line, we add a periodic boundary condition on both sides of line, and subsequently computes the dispersion curve using the Eigenmode Solver of CST Microwave Studio. The scattering parameters are measured using an Agilent network analyzer PNA E5071C. We use the commercial measurement system Satimo Starlab to measure the near-field performance and then numerically compute the far-field radiation patterns.
